# Case report: Clinical features and management outcomes of isolated corneal intraepithelial neoplasia

**DOI:** 10.3389/fopht.2024.1346361

**Published:** 2024-01-31

**Authors:** Samar A. Al-Swailem, Hind M. Alkatan, Huda Saif AlDhaheri, Sara M. AlHilali, Azza M. Y. Maktabi

**Affiliations:** ^1^ Anterior Segment Division, King Khaled Eye Specialist Hospital, Riyadh, Saudi Arabia; ^2^ Ophthalmology Department, College of Medicine, King Saud University, Riyadh, Saudi Arabia; ^3^ Ophthalmology Department, Tawam Hospital, Al Ain, United Arab Emirates; ^4^ Pathology and Laboratory Medicine, King Khaled Eye Specialist Hospital, Riyadh, Saudi Arabia

**Keywords:** squamous cell carcinoma, ocular surface squamous neoplasia, stem cell deficiency, interferon, mitomycin C

## Abstract

**Purpose:**

To report clinical features and treatment outcome of three cases with isolated corneal intraepithelial neoplasia (CIN).

**Methods:**

This case series presents 3 patients with isolated CIN. Data collected included, presenting signs and symptoms including vision, anterior segment examination, medical and surgical outcomes and signs and symptoms at lost post-treatment visit.

**Results:**

Case 1 was a 45-year-old male who presented with an isolated grayish amoeboid corneal lesion which was excised with alcohol assisted epitheliectomy, he also received 6 cycles of topical mitomycin C (MMC) 0.02% and one injection of interferon alfa-2b with no recurrence during the 10-year follow-up period. Case 2 was 78-year-old male referred for a suspicious white corneal lesion which was completely excised, the patient also received 6 subconjunctival injections of interferon alpha-2b. However, the lesion recurred at 2.5-years post-treatment. Case 3 was a 63-year-old male patient who presented with an isolated corneal lesion that was excised using alcohol-assisted epitheliectomy, patient received four cycles of topical 5-fluorouracil with no recurrence at last follow-up visit at 6 months.

**Conclusion:**

Isolated corneal intraepithelial neoplasia (CIN) is a rare entity with few reported cases in the literature. In this case series, we report long and short-term management outcomes of combined surgical and medical therapy for isolated CIN.

## Introduction

Ocular surface squamous neoplasia (OSSN) predominantly affects the limbus, conjunctiva, and cornea, with isolated corneal intraepithelial neoplasia (CIN) being relatively rare (1). Isolated CIN is characterized by the presence of neoplastic cells confined to the corneal epithelium, which poses diagnostic and therapeutic challenges (2). Its clinical presentation often overlaps with other benign and malignant ocular surface conditions, necessitating a high index of suspicion and thorough investigation. *In vivo* confocal microscopy and anterior segment optical coherence tomography are valuable diagnostic adjuncts, complementing the gold standard of histopathological examination (3).

The management of OSSN has evolved significantly over time, with a shift toward conservative approaches such as topical chemotherapy and immunomodulatory agents (4). These treatment modalities have demonstrated promising results, offering better ocular surface preservation, and minimizing surgical complications (4). This case series reports the clinical features, diagnosis and treatment outcomes of three eyes with isolated CIN.

## Case reports

### Case 1

**Figure 1:**
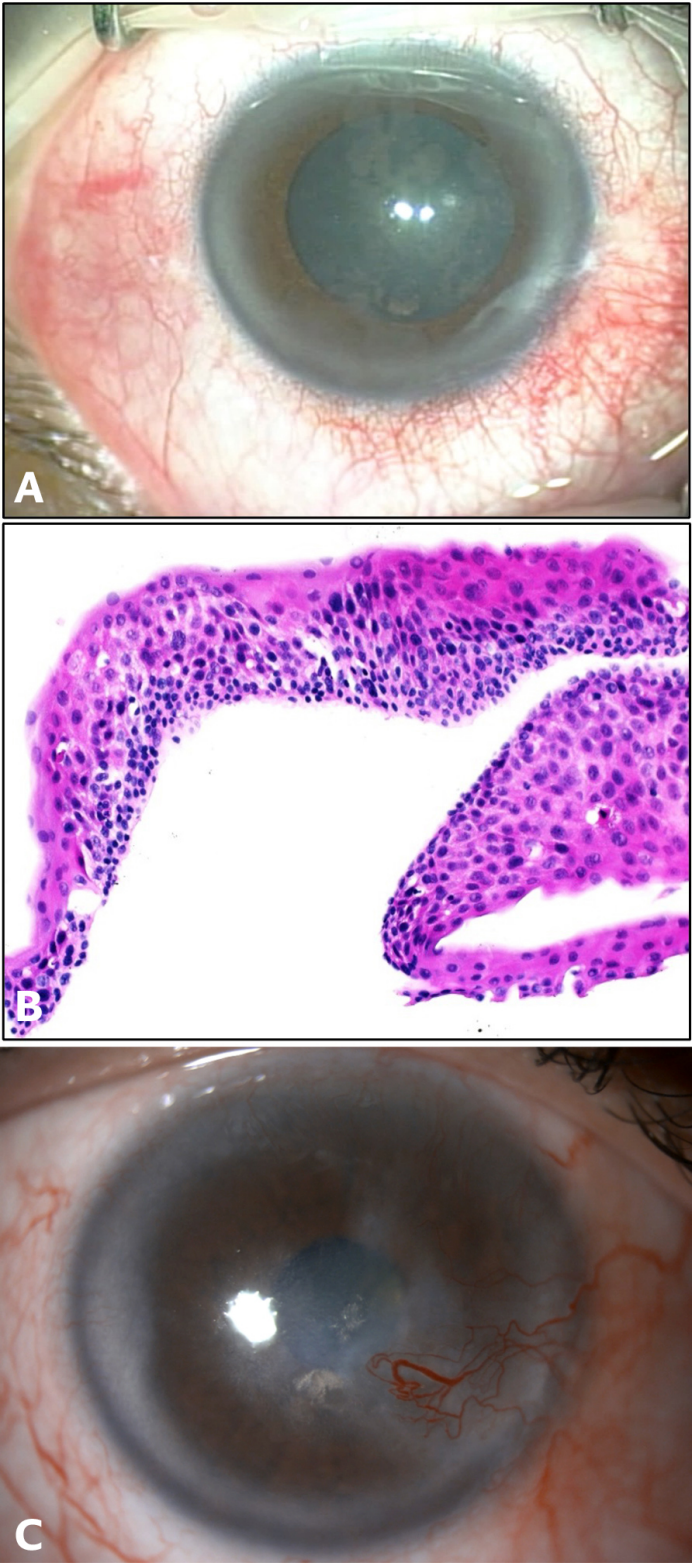
Case 1: **(A)** Intraoperative image of the right eye showing grayish amoeboid epithelial abnormality obstructing the visual axis. **(B)** Histopathology slide with hematoxylin and eosin (H&E) stain showing increased epithelial thickness with dysplastic features including pleomorphism, prominent nucleoli and loss of polarity, intact underlying basement membrane. Features consistent with corneal intraepithelial neoplasia. **(C)** Slit-lamp image of the right eye at the final follow-up visit.

A 45-year-old male presented with gradually worsening vision in his right eye over the course of one year, accompanied by a persistent foreign body sensation. Upon examination, his best-corrected visual acuity (BCVA) in the right eye was 20/60. Biomicroscopy of the cornea revealed a central, grayish, amoeboid epithelial irregularity, measuring approximately 6 mm horizontally and 8 mm vertically (**Figure 1A**).

The lesion was completely excised using alcohol-assisted epitheliectomy. Histopathological examination confirmed the diagnosis of CIN. The tumor cells displayed characteristics such as anaplasia, increased mitotic activity, loss of polarity, and pleomorphism (**Figure 1B**).

Once the epithelium was completely healed, the patient commenced treatment with topical 0.02% mitomycin C (MMC) for a total of six cycles, each consisting of two weeks on and two weeks off. After completing the sixth cycle, the patient developed a large epithelial defect (ED) along with severe ocular discomfort. MMC was discontinued, and extensive lubrication was started, the ED did heal however, MMC resulted in localized limbal stem cell deficiency (LSCD). The patient received a single subconjunctival injection of pegylated interferon alpha-2b (80 mcg/0.5 cc). At the last follow-up visit, ten-years post-treatment, the patient’s BCVA was 20/200, with no reported recurrence of the lesion (**Figure 1C**).

### Case 2

**Figure 2:**
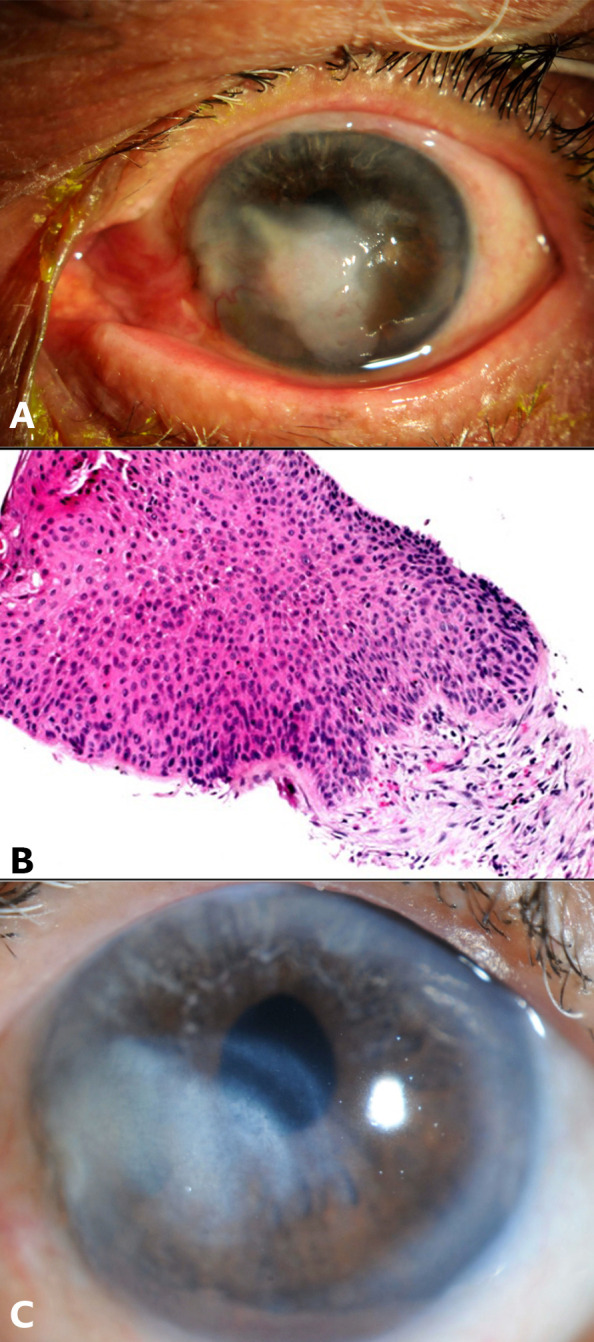
Case 2: **(A)** Slit-lamp image of the left eye showing central whitish elevated corneal lesion with superficial blood vessels. **(B)** Histopathology slide with hematoxylin and eosin (H&E) stain showing increased epithelial thickness with dysplastic features including pleomorphism, prominent nucleoli and loss of polarity, intact underlying basement membrane. Features consistent with corneal intraepithelial neoplasia. **(C)** Slit-lamp image of the left eye at the final follow-up visit.

A 78-year-old male presented with a history of gradually declining vision in his left eye over a period of five months. Upon examination, BCVA was 4/200 in the left eye. Biomicroscopy revealed a white, elevated lesion in the cornea measuring approximately 5 mm horizontally and 5 mm vertically (**Figure 2A**).

The patient underwent superficial keratectomy with application of 20% alcohol for 30 seconds post-excision. Histopathological examination confirmed the diagnosis of CIN (**Figure 2B**).

The patient received six subconjunctival injections of pegylated interferon alpha-2b (80 mg/0.5 cc) over a period of 4 months. He reported experiencing flu-like symptoms, which subsequently improved with the administration of oral non-steroidal anti-inflammatory medications. At the 2.5-years follow-up visit, the patient presented with recurrence of the corneal epithelial lesion at the same site, measuring 5 mm horizontally and 3 mm vertically. The patient underwent alcohol-assisted epitheliectomy resulting in the complete excision of the lesion. Histopathological examination confirmed the recurrence of CIN with severe dysplasia. Postoperatively, the patient received topical 5-fluorouracil (5-FU) four times daily, using a regimen of two weeks on and two weeks off for a total of four cycles, with topical fluorometholone (FML) administered during the off periods. At the last follow-up appointment, five-years post-treatment, the BCVA was 20/40 there were no reported recurrences (**Figure 2C**).

### Case 3

**Figure 3:**
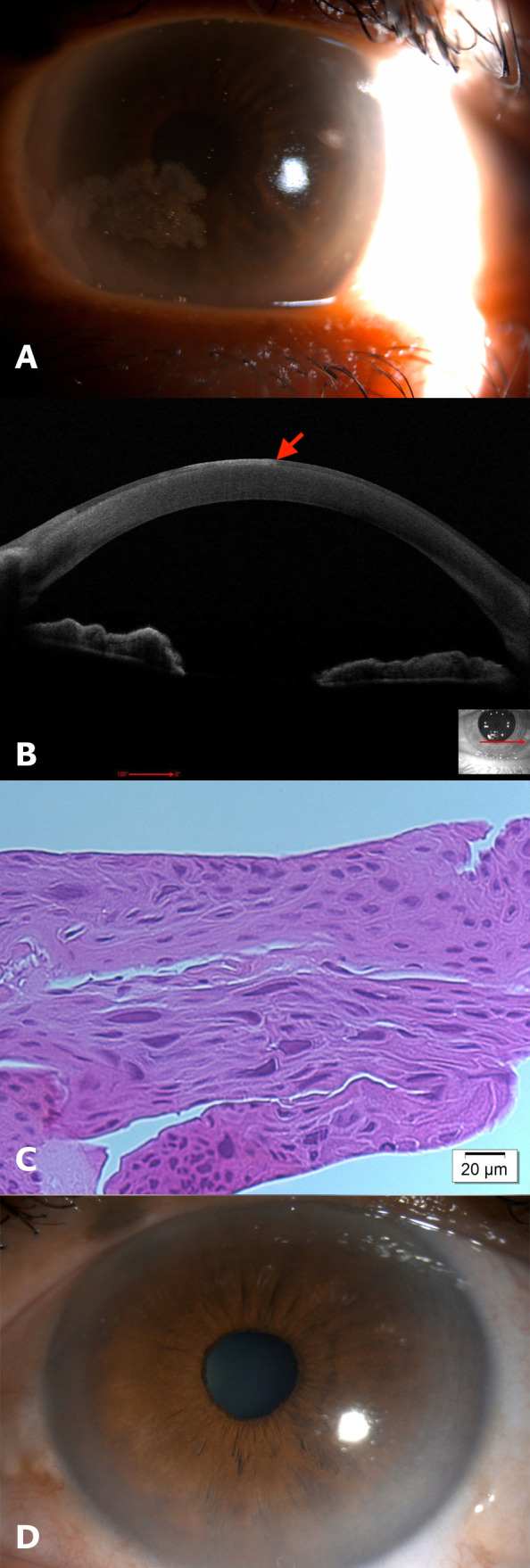
Case 3: **(A)** Slit-lamp image of the left eye showing inferonasal greyish epithelial lesion with amoeboid appearance involving the visual axis. **(B)** Anterior segment optical coherence tomography (AS-OCT) of the left eye showing hyperreflective epithelium with abrupt transition between normal and abnormal epithelium (red arrow). **(C)** Histopathology slide with hematoxylin and eosin (H&E) stain showing increased epithelial thickness with dysplastic features including pleomorphism, prominent nucleoli and loss of polarity. Features consistent with corneal intraepithelial neoplasia. **(D)** Slit-lamp image of the left eye at the final follow-up visit.

A 63-year-old male presented to the emergency department reporting mild irritation and a scratchy sensation in his left eye that had persisted for four months. Upon examination, the BCVA in the left eye was 20/25. Slit lamp examination of the left cornea revealed a grayish, raised epithelial lesion with fimbriated margins, measuring approximately 6 mm horizontally and 2 mm vertically. (**Figure 3A**) Anterior segment optical coherence tomography (AS-OCT) confirmed the presence of hyperreflective epithelial lesion with abrupt transition between normal and abnormal epithelium at the margin of the lesion (**Figure 3B**).

The patient underwent alcohol-assisted epithelial removal under topical anesthesia to completely excise the lesion. Histopathological examination of the excised tissue confirmed severe epithelial dysplasia, consistent with carcinoma in situ (**Figure 3C**).

Following the procedure, the patient received topical 5-FU four times daily, using a regimen of two weeks on and two weeks off for a total of four cycles, with topical FML administered during the off periods. At the last follow-up visit, six-months post-treatment, there were no signs of recurrence and the BCVA was 20/20 in the left eye (**Figure 3D**).

## Discussion

Isolated corneal OSSN is a rare clinical diagnosis, with few case reports available in the literature (2, 5–12). Published reports from the past 20 years are summarized in **Table 1**. The corneoscleral limbus demonstrates increased mitotic activity and is almost always implicated in dysplastic lesions of the cornea or conjunctiva (2). In our patients, the corneoscleral limbus was spared, potentially supporting the theory that isolated corneal squamous epithelial neoplasia may result from the centripetal migration of abnormal epithelial cells from the limbus, which later develop malignant potential within central corneal regions (7). High resolution anterior segment optical coherence tomography (AS-OCT) can aid in the diagnosis of isolated CIN (3). High resolution AS-OCT is a non-invasive imaging modality that provides an optical biopsy of the CIN lesions. It was also confirmed that OCT images show excellent correlation with histopathological specimens of eyes with CIN (3).

**Table 1:** Features of reported cases with isolated corneal squamous cell carcinoma [1991-2021].

Reference	Year	Country	Age	Sex	History	Appearance	Histopathology	Treatment	Follow up and recurrence
**Cameron et al. (** 7)	1991	KSA	40	M	Pain and blood -Tinged discharge for 3 weeks	Elevated central lesion 10mm width and 8 mm in height with superficial blood vessels and keratinization	Microinvasive SCC	Superficial keratectomy + Cryotherapy	46 months – None
			57	M	Decreased vision and whitish spot for 1 year	Central, elevated bilobed white corneal lesion with superficial blood vessels and keratinization	Invasive SCC	PKP + ECCE + PC IOL	9 months – None
**Shin et al. (** 6)	2001	Korea	73	M	Whitish mass for 15 days	Elevated fixed nodular mass at the interpalpebral region, hyper- pigmented patch with superficial blood vessels	Well differentiated SCC with 1mm invasion beyond basement membrane	Wide surgical excision + Cryotherapy	11 years – None
**Arya et al. (** 2)	2008	India	80	M	Decreased vision, tearing and foreign body sensation for 1 year	White central, elevated gelatinous mass (7mm x6mm) with feeder superficial blood vessels	Well differentiated invasive SCC	Surgical excision + MCC 0.002% for 4 weeks	6 months – None
**Khan et al. (** 8)	2013	India	35	M	Foreign body sensation and tearing for 3 months.	Whitish, pink, fixed nodular fungating mass at temporal side of cornea (3mm).	SCC	Wide surgical excision + Cryotherapy	6 months – None
**Chin et al. (** 11)	2013	USA	57	M	Blurry vision for 2 months	Translucent corneal epithelial lesion	CIN with no invasion of the basement membrane	Superficial keratectomy + topical INF-a2b for 3 months	6 months – None
**Ganavati et al. (** 5)	2014	Iran	48	M	Blurry vision and dry eye	Epitheliopathy and filamentary keratopathy.	CIN	Debridement + two cycles of topical 5-FU (2 weeks on and 1 week off)	45 days – None
**Morii et al. (** 10)	2016	Japan	76	M	Visual disturbance	Whitish elevated plaque-like corneal lesion	Atypical dysplastic epithelial cells	Superficial keratectomy	7 months – None
**Marticorena-Alvarez et al. (** 12)	2020	Spain	80	F	Foreign body sensation	Well demarcated grey corneal plaque	High-grade epithelial neoplasia	Debridement followed by application of alcohol + AMT, Post-op started on INF-a2b for 1 month	3 years – None
**Singh et al. (** 9)	2021	India	67	M	Progressively enlarging corneal mass	Central corneal lesion measuring 7.5mm*4mm with whitish avascular plaque-like appearance	Keratinizing hyperplastic epithelium with moderate dysplasia	Superficial epitheliectomy + AMT	Had recurrence at 6 weeks, treated with topical INF-a2b for 2 months 6 months FU – no recurrence

*KSA, Kingdom of Saudi Arabia; USA, United States of America; M, Male; F, Female; mm, millimeter; SCC, Squamous cell carcinoma; CIN, Corneal Intraepithelial Neoplasia; PKP, Penetrating Keratoplasty; ECCE, Extracapsular cataract extraction; PC IOL, posterior chamber intraocular lens; MMC, mitomycin C; AMT, amniotic membrane transplantation; INF-a2b, Interferon alpha 2b.

Due to the rarity of isolated CIN, there is no clear consensus or evidence-based data on the optimal treatment guideline for this condition. However, various treatment modalities have been reported in the literature, with the primary goal of complete tumor removal and preservation of ocular function (4). Superficial keratectomy is the most commonly performed surgical intervention for isolated CIN. It involves the removal of the abnormal corneal epithelium, leaving the underlying Bowman’s layer and stroma intact (2, 6). Mitomycin C (MMC) is a commonly used topical chemotherapeutic agent for treating OSSN, including isolated CIN (4). Topical chemotherapeutics are usually applied as eye drops in a cyclic manner (e.g., two weeks on, two weeks off) and can be used as an adjunct to surgical excision or as a primary treatment in select cases (4). Limbal stem cell deficiency (LSCD) remains a debilitating side-effect of MMC, as reported in case 1, the patient developed localized LSCD and corneal epitheliopathy, which contributed to his reduced BCVA at the last follow-up visit. Therefore, it is perhaps better to avoid MMC as first line therapy, especially in cases of isolated CIN. Topical 5-fluorouracil (5-FU) is another chemotherapeutic option for treating OSSN with a similar regimen of MMC (4). Long-term treatment outcomes of topical 5-FU have been reported with promising safety and efficacy profiles (4). Furthermore, we have reported successful treatment of the recurrent lesion of case 2 with topical 5-FU, which may suggest that 5-FU is an effective treatment option for recurrent CIN. However, further studies are warranted to confirm this finding.

Interferon alpha-2b has shown promise as an adjuvant treatment for OSSN (4). It can be administered either topically or through subconjunctival injections. Interferon therapy may offer a favorable safety profile compared to other treatments, with fewer ocular surface-related side effects (4). A group from Spain have reported the complete resolution of conjunctival squamous neoplasia with the use of topical interferon alpha 2b for 1 month deferring the need for surgery (13). Resistance to chemotherapy remains a significant challenge in cancer treatment, there are few attributing factors such as genetic mutations in cancer cells that make them inherently resistant or drug exposure that leads to acquired resistance (14). A case series of 3 immunosuppressed patients with OSSN reported poor response to topical interferon-alpha-2b therapy, the authors postulated immune suppression as a risk factor to interferon treatment failure (15).

In conclusion, isolated CIN is a rare subset of OSSN. Given the rarity of this condition, it is essential for clinicians to be vigilant in recognizing the clinical features of this condition to ensure timely and appropriate intervention. Superficial keratectomy remains the mainstay of treatment with or without adjunct chemotherapy. 5-FU might be an effective treatment option for recurrent isolated CIN. Future research should focus on conducting larger studies and case series to better understand the etiology, risk factors, and optimal treatment strategies for isolated CIN.

## Data availability statement

The original contributions presented in the study are included in the article/supplementary material. Further inquiries can be directed to the corresponding author.

## Ethics statement

The studies involving humans were approved by Institutional Research Board (KKESH, RP 1577-R) at King Khaled Eye Specialist Hospital. The studies were conducted in accordance with the local legislation and institutional requirements. The participants provided their written informed consent to participate in this study. Written informed consent was obtained from the individual(s) for the publication of any potentially identifiable images or data included in this article.

## Author contributions

SA-S: Conceptualization, Supervision, Writing – original draft, Writing – review & editing. HSA: Writing – original draft. HA: Writing – review & editing, Data curation, Investigation, Supervision. SA: Writing – review & editing, Writing – original draft. AM: Investigation, Writing – review & editing.
